# Mycotoxin Zearalenone Attenuates Innate Immune Responses and Suppresses NLRP3 Inflammasome Activation in LPS-Activated Macrophages

**DOI:** 10.3390/toxins13090593

**Published:** 2021-08-25

**Authors:** Po-Yen Lee, Ching-Chih Liu, Shu-Chi Wang, Kai-Yin Chen, Tzu-Chieh Lin, Po-Len Liu, Chien-Chih Chiu, I-Chen Chen, Yu-Hung Lai, Wei-Chung Cheng, Wei-Ju Chung, Hsin-Chih Yeh, Chi-Han Huang, Chia-Cheng Su, Shu-Pin Huang, Chia-Yang Li

**Affiliations:** 1 Graduate Institute of Medicine, College of Medicine, Kaohsiung Medical University, Kaohsiung 80708, Taiwan; maco69@gmail.com (P.-Y.L.); retina.liu@gmail.com (C.-C.L.); joice715@gmail.com (K.-Y.C.); 990327kmuh@gmail.com (T.-C.L.); yljane.chen@gmail.com (I.-C.C.); yuhung.lai@gmail.com (Y.-H.L.); chung.wj69@gmail.com (W.-J.C.); cheryl60286@gmail.com (C.-H.H.); s940854@gmail.com (C.-C.S.); 2 Department of Ophthalmology, Kaohsiung Medical University Hospital, Kaohsiung Medical University, Kaohsiung 80708, Taiwan; 3 Department of Ophthalmology, Chi Mei Medical Center, Tainan 71004, Taiwan; 4 Department of Medical Laboratory Science and Biotechnology, Kaohsiung Medical University, Kaohsiung 80708, Taiwan; shuchiwang@kmu.edu.tw; 5 Department of Internal Medicine, Division of Cardiology, Kaohsiung Medical University, Kaohsiung 80708, Taiwan; 6 Department of Respiratory Therapy, College of Medicine, Kaohsiung Medical University, Kaohsiung 80708, Taiwan; kisa@kmu.edu.tw; 7 Department of Biotechnology, Kaohsiung Medical University, Kaohsiung 80708, Taiwan; cchiu@kmu.edu.tw; 8 Department of Pediatrics, Kaohsiung Medical University Hospital, Kaohsiung 80756, Taiwan; 9 Department of Pediatrics, School of Medicine, College of Medicine, Kaohsiung Medical University, Kaohsiung 80708, Taiwan; 10 Department of Ophthalmology, School of Medicine, College of Medicine, Kaohsiung Medical University, Kaohsiung 80708, Taiwan; 11 Research Center for Tumor Medical Science, Graduate Institute of Biomedical Sciences, China Medical University, Taichung 40402, Taiwan; cwc0702@gmail.com; 12 Ph.D. Program for Cancer Biology and Drug Discovery, China Medical University and Academia Sinica, Taichung 40402, Taiwan; 13 Department of Urology, School of Medicine, College of Medicine, Kaohsiung Medical University, Kaohsiung 80708, Taiwan; patrick1201.tw@yahoo.com.tw (H.-C.Y.); shpihu73@gmail.com (S.-P.H.); 14 Department of Urology, Kaohsiung Municipal Ta-Tung Hospital, Kaohsiung 80145, Taiwan; 15 Department of Surgery, Division of Urology, Chi-Mei Medical Center, Tainan 71004, Taiwan; 16 Department of Senior Citizen Service Management, Chia Nan University of Pharmacy and Science, Tainan 71710, Taiwan; 17 Department of Urology, Kaohsiung Medical University Hospital, Kaohsiung Medical University, Kaohsiung 80708, Taiwan; 18 Graduate Institute of Clinical Medicine, College of Medicine, Kaohsiung Medical University, Kaohsiung 80708, Taiwan; 19 Department of Medical Research, Kaohsiung Medical University Hospital, Kaohsiung 80756, Taiwan

**Keywords:** zearalenone, mycotoxin, innate immunity, NLRP3 inflammasome, macrophages

## Abstract

Zearalenone (ZEA) is a mycotoxin that has several adverse effects on most mammalian species. However, the effects of ZEA on macrophage-mediated innate immunity during infection have not been examined. In the present study, bacterial lipopolysaccharides (LPS) were used to induce the activation of macrophages and evaluate the effects of ZEA on the inflammatory responses and inflammation-associated signaling pathways. The experimental results indicated that ZEA suppressed LPS-activated inflammatory responses by macrophages including attenuating the production of proinflammatory mediators (nitric oxide (NO) and prostaglandin E_2_ (PGE_2_)), decreased the secretion of proinflammatory cytokines (tumor necrosis factor (TNF)-α, interleukin (IL)-1β and IL-6), inhibited the activation of c-Jun amino-terminal kinase (JNK), p38 and nuclear factor-κB (NF-κB) signaling pathways, and repressed the nucleotide-binding and oligomerization domain (NOD)-, leucine-rich repeat (LRR)- and pyrin domain-containing protein 3 (NLRP3) inflammasome activation. These results indicated that mycotoxin ZEA attenuates macrophage-mediated innate immunity upon LPS stimulation, suggesting that the intake of mycotoxin ZEA-contaminated food might result in decreasing innate immunity, which has a higher risk of adverse effects during infection.

## 1. Introduction

Zearalenone (ZEA), a non-steroidal estrogenic mycotoxin, is produced by several species of Fusarium fungi that widely contaminate many cereal crops including wheat, corn, sorghum, oats, and barley, and subsequently produce ZEA at low temperatures and high humidity environments [1]. The European Food Safety Authority (EFSA) established a tolerable daily intake (TDI) for ZEA at 0.25 µg/kg body weight in 2011 [2]. While ZEA exhibits low acute toxicity (oral LD_50_ > 2000 mg/kg body weight [3]), long-term exposure to ZEA has several harmful effects due to its toxicity and high estrogenic activity including immunotoxic [4], hepatotoxic [5], and genotoxic [6] effects; however, the effect of ZEA on regulation of immune responses has not been well evaluated.

The innate immunity acts as the first line of defense against pathogen infection, and macrophages are antigen-presenting cells in the innate immune system that can phagocytose bacteria and produce both proinflammatory cytokines (e.g., tumor necrosis factor-α (TNF-α), interleukin (IL)-1β and IL-6), and mediators (e.g., nitric oxide (NO) and cyclooxygenase-2 (COX-2, a key enzyme in the synthesis of prostaglandins)) [7]. Moreover, macrophages can also present antigens to T cells and act as effectors for the induction of adaptive immune responses. Macrophages recognize pathogen-associated molecular patterns (e.g., lipopolysaccharide (LPS)) and damage-associated molecular patterns (e.g., adenosine triphosphate (ATP) and nigericin) by pathogen recognition receptors (e.g., toll-like receptors (TLRs)) and subsequently activate the downstream mitogen-activated protein kinases (MAPKs, such as the c-Jun amino-terminal kinase (JNK), the extracellular signal-regulated protein kinase (ERK), the p38 MAP kinase (p38)) and transcription factors (e.g., nuclear factor-κB (NF-κB)) to regulate immune responses against pathogen infection [8,9].

Inflammasomes are cytosolic protein complexes that modulate caspase-1 activation in innate immune responses and subsequently process both IL-1β and IL-18 maturation and secretion [10]. Nucleotide-binding and oligomerization domain (NOD)-, leucine-rich repeat (LRR)- and pyrin domain-containing protein 3 (NLRP3) inflammasome, the best-characterized inflammasome, can be activated by many stimuli, including extracellular adenosine triphosphate (ATP), pore-forming toxins, mitochondrial reactive oxygen species (ROS), potassium efflux, and destabilized lysosomes [11]. The activation of NLRP3 inflammasomes are central to elicit innate immune responses and are crucial for host immune response to bacterial [12], fungal [13], and viral infections [14,15]. Defects of inflammasome activation have been demonstrated to increased bacterial burden in systemic organs like the liver, lung, and spleen [16].

Although several reports have indicated that ZEA has either stimulating or suppressing effects on innate immunity [17,18], the cellular mechanisms activated by ZEA in triggering innate immune responses in macrophages during pathogen infection are not yet well understood. The present study investigated the effects of ZEA on the activities of innate immune responses during pathogen infection by macrophages in vitro and ex vivo. LPS was used as stimulus to mimic bacterial infection, which can trigger innate immune responses by macrophages. The secretions of proinflammatory cytokines and mediators, NLRP3 inflammasome activation, and the activities of both MAPKs and NF-κB signaling pathways were examined.

## 2. Results

### 2.1. ZEA Attenuates Inducible Nitric Oxide Synthase (iNOS) and COX-2 Expressions of and Inhibits NO and Prostaglandin E2 (PGE_2_) Productions by LPS-Activated Macrophages

**Figure 1:**
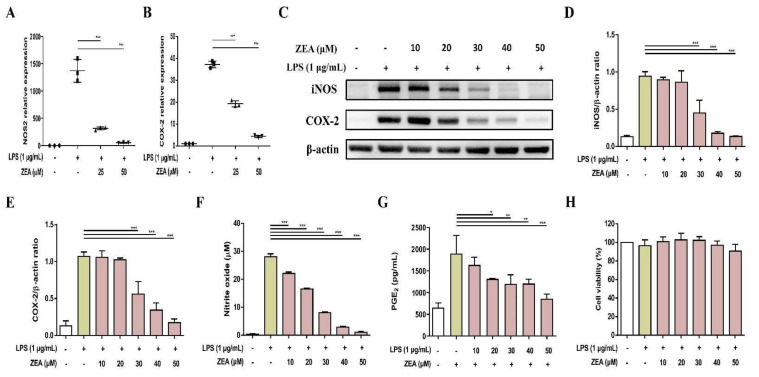
The effect of ZEA on the inflammatory mediator productions by LPS-activated macrophages. J774A.1 cells were pre-treated with ZEA for 1 h, and then treated with 1 μg/mL LPS for 6 (for qPCR) and 24 h (for Griess reaction, Western blot, ELISA and MTT assay). (**A**,**B**) The gene expression of *NOS2* and *COX-2* was measured using qPCR (n = 5). The expression of iNOS and COX-2 was detected by Western blot. The representative images are shown in (**C**), and the quantified results from three independent experiments in (**D**,**E**). (**F**) The levels of NO production were analyzed using Griess reaction (**G**) The secretion of PGE_2_ was analyzed by ELISA. (**H**) Cell viability was examined using MTT assay. Data from three separate experiments are presented as mean ± standard deviation (SD). Statistical significances are presented as * *p* < 0.05; ** *p* < 0.01; *** *p* < 0.001.

In response to pathogen infection, NO is an important proinflammatory mediator secreted by activated macrophages, which are produced by enzyme iNOS [19]. The levels of NO and iNOS are associated with COX-2 expression, which is an essential enzyme converting arachidonic acid to PGE_2_, one of the main pro-inflammatory factors [20,21]. LPS was used as a stimulus to mimic bacterial infection for macrophage activation. The effect of mycotoxin ZEA on the secretion of NO and PGE_2_ was determined using Griess reaction and enzyme-linked immunosorbent assay (ELISA) respectively, while the expression of iNOS and COX-2 was determined by quantitative polymerase chain reaction (qPCR) and Western blot. The experimental results pointed out that ZEA suppressed the iNOS and COX-2 expressions in both mRNA and protein levels (Figure 1A–E), inhibited the production of NO (Figure 1F), and reduced the secretion of PGE_2_ (Figure 1G) in LPS-activated J774A.1 cells. To exclude the potential cytotoxic effect of ZEA on LPS-activated inflammatory responses by macrophages, the effect of ZEA on the cell viability was detected by 3-[4,5-dimethylthiazol-2-yl]-2,5 diphenyl tetrazolium bromide (MTT) assay. The results pointed out that the doses of ZEA ≤ 50 μM did not affect the viability of LPS-activated J774.1 cells (Figure 1H), indicating that ZEA exhibited inhibitory effects on LPS-activated inflammatory responses by macrophages.

### 2.2. ZEA Suppresses the Expression and Production of TNF-α and IL-6 by LPS-Activated Macrophages

**Figure 2:**
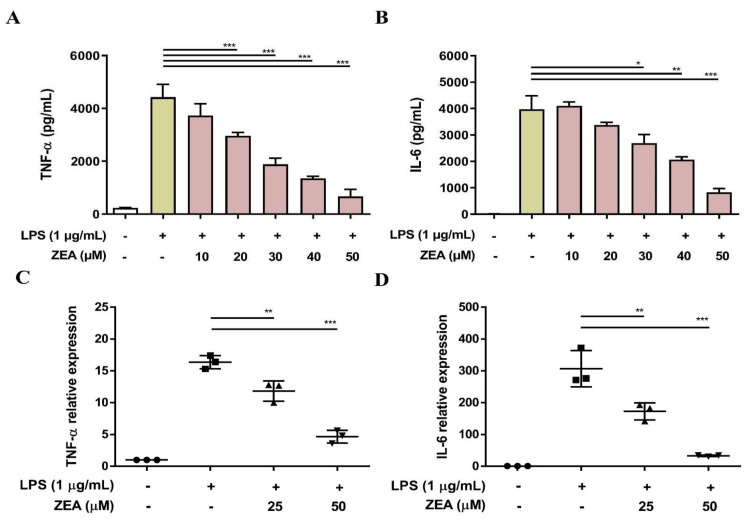
The effect of ZEA on the secretion of proinflammatory cytokines by LPS-activated macrophages. J774A.1 cells were pre-treated with ZEA for 1 h, and then treated with 1 μg/mL LPS for 6 (for qPCR) and 24 h (for ELISA). (**A**,**B**) The secretion of TNF-α and IL-6 was determined using ELISA. Data from three separate experiments are presented as mean ± SD. (**C**,**D**) The gene expression of *TNF-α* and *IL-6* was measured by qPCR (n = 5). Statistical significances are presented as * *p* < 0.05; ** *p* < 0.01; *** *p* < 0.001.

Both TNF-α and IL-6 are critical proinflammatory cytokines against pathogen infection, and a lack of TNF-α and IL-6 results in higher mortality and more susceptibility to bacterial infection [22,23]. To examine whether ZEA affects LPS-activated TNF-α and IL-6 production by macrophages, J774A.1 cells were pretreated with ZEA for 1 h, and then treated with 1 μg/mL LPS for 6 or 24 h. The secretion of TNF-α and IL-6 was analyzed by ELISA and the gene expression of *TNF-α* and *IL-6* was measured by qPCR. The experimental results indicated that ZEA significantly attenuated the secretion of TNF-α and IL-6 by LPS-activated macrophages (Figure 2A,B). In addition, ZEA also suppressed the expression of *TNF-α* and *IL-6* by LPS-activated J774A.1 cells (Figure 2C,D).

### 2.3. ZEA Inhibits the Activation of MAPKs and NF-κB Signaling Pathways by LPS-Activated Macrophages

**Figure 3:**
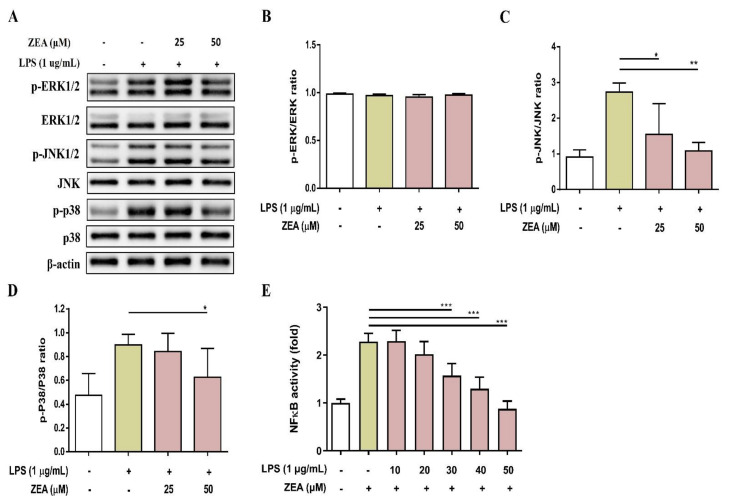
The effect of ZEA on the expression of MAPK signaling cascades-associated protein and the promoter activity of NF-κB by LPS-activated macrophages. J774A.1 cells were pretreated with ZEA for 1 h, and then treated with 1 μg/mL LPS for 2 h. The phosphorylation and expression of ERK, JNK and p38 were detected by Western blot. The expression of β-actin was used as loading control. The representative images are shown in (**A**), and the quantified results from three independent experiments shown in (**B**–**D**). (**E**) J-blue cells were pretreated with ZEA for 1 h, and then treated with 1 μg/mL LPS for 24 h. The level of secreted embryonic alkaline phosphatase (SEAP) was examined. Data from three separate experiments are presented as mean ± SD. Statistical significances are presented as * *p* < 0.05; ** *p* < 0.01; *** *p* < 0.001.

MAPKs and NF-κB signaling are two critical pathways downstream of TLRs that drive inflammatory responses during infection [24]. To examine whether ZEA affects LPS-activated MAPKs and NF-κB signaling pathways by macrophages, J774A.1 cells were pretreated with ZEA for 1 h, and then treated with 1 μg/mL LPS for 2 or 24 h. These activations were measured by Western blot and promoter reporter assay respectively. As shown in Figure 3A–D, ZEA significantly attenuated the phosphorylation of JNK and p38 by LPS-activated macrophages, but not ERK. Moreover, ZEA was found to significantly decrease the promoter reporter activity of NF-κB by LPS-activated macrophages (Figure 3E).

### 2.4. ZEA Inhibits IL-1β Secretion and Suppresses NLRP3 Inflammasome Activation by LPS/ATP- and LPS/nigericin-Activated Macrophages

**Figure 4:**
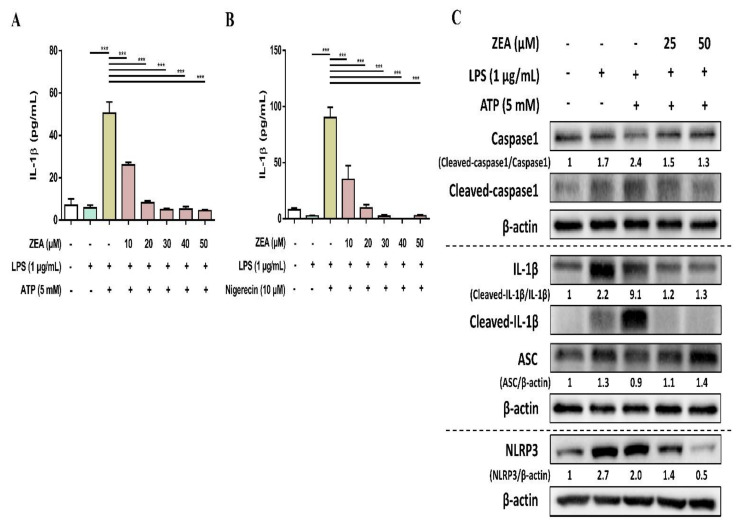
The effect of ZEA on the activation of NLRP3 inflammasome by LPS-activated macrophages. J774A.1 cells were pretreated with ZEA for 1 h, and then treated with 1 μg/mL LPS for 5 h following 5 mM ATP or 10 μΜ nigericin treatments for 30 min. (**A**,**B**) The secretion of IL-1β was analyzed by ELISA. Data from three separate experiments are presented as mean ± SD. NLRP3 inflammasome-associated protein expressions were determined by Western blot. A representative image of three independent experiments is shown in (**C**). Statistical significances are presented as *** *p* < 0.001.

**Figure 5:**
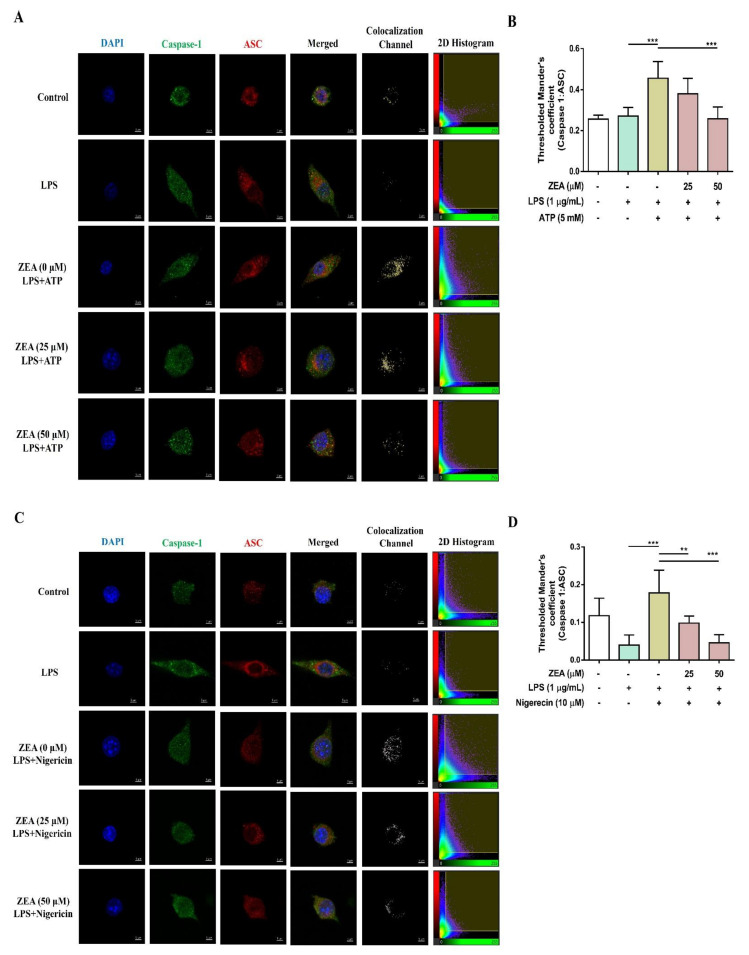
The effect of ZEA on the formation of NLRP3 inflammasome by LPS-activated macrophages. J774A.1 cells were pretreated with ZEA for 1 h, and then treated with 1 μg/mL LPS for 5 h following ATP (5 mM) or nigericin (10 μΜ) treatments for 30 min. (**A**,**C**) Representative images of colocalization of caspase-1 (green) with ASC (red) were shown. DAPI was used as a nuclear counterstain. (**B**,**D**) The formation of ASC speck was quantified using the colocalization of caspase-1 and ASC signals in the threshold of 2D histogram (in the panel **A** and **C**) by Mander’s coefficient. Data from three separate experiments are presented as mean ± SD. Statistical significances are presented as ** *p* < 0.01; *** *p* < 0.001.

The activation of NLRP3 inflammasome by microbial stimuli plays a critical role in regulating IL-1β and IL-18 secretion during infection [25]. To examine whether ZEA affects the activation of NLRP3 inflammasome by LPS/ATP- and LPS/nigericin-activated macrophages, J774A.1 cells were pretreated with ZEA for 1 h, and then treated with 1 μg/mL LPS for 5 h following 5 mM ATP or 10 μM nigericin treatments for 30 min. IL-1β secretion, NLRP3 inflammasome-associated protein expressions, and ASC and caspase-1 colocalization were analyzed by ELISA, Western blot, and immunofluorescence staining. Our experimental results showed that ZEA significantly suppressed IL-1β secretion of by LPS/ATP- and LPS/nigericin-activated macrophages (Figure 4A,B). ZEA also inhibited the expression of NLRP3, the cleavage of pro-caspase-1 to cleaved caspase-1 and the cleavage of pro-IL-1β to cleaved IL-1β by LPS/ATP-activated macrophages (Figure 4C). Moreover, ZEA inhibited ASC and caspase-1 colocalization in LPS/ATP- and LPS/nigericin-activated macrophages (Figure 5), indicating the ZEA inhibited the NLRP3 inflammasome assembly.

### 2.5. ZEA Suppresses Proinflammatory Cytokine Secretions by LPS-Activated Human Macrophages

**Figure 6:**
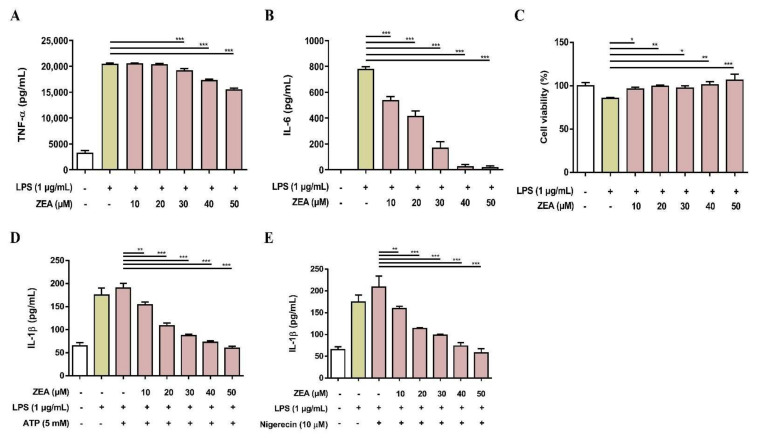
The effect of ZEA on the proinflammatory cytokine production by LPS-activated human monocyte-derived macrophages. THP-1 cells were stimulated with 50 ng/mL PMA for 24 h to induce macrophage differentiation. Afterwards, cells were pretreated with ZEA for 1 h following 1 μg/mL LPS treatment for 24 h. The secretion of (**A**) TNF-α and (**B**) IL-6 was detected by ELISA. (**C**) Cell viability was measured using MTT assay. (**D**,**E**) Human monocyte-derived macrophages were pretreated with ZEA for 1 h, and then treated with 1 μg/mL LPS for 5 h following ATP (5 mM) or nigericin (10 μΜ) treatments for 30 min. The secretion of IL-1β was examined using ELISA. Data from three separate experiments are presented as mean ± SD. Statistical significances are presented as * *p* < 0.05; ** *p* < 0.01; *** *p* < 0.001.

To examine whether ZEA affects LPS-activated proinflammatory cytokine secretions by human macrophages, THP-1 cells were induced to undergo macrophage differentiation by phorbol 12-myristate 13-acetate (PMA) for 24 h and then pretreated with ZEA for 1 h following 1 μg/mL LPS treatment for 24 h. The secretion of TNF-α and IL-6 was analyzed by ELISA. Cell viability was measured by MTT assay. As shown in Figure 6A,B, ZEA suppressed LPS-activated TNF-α and IL-6 secretions by human macrophages. In addition, the doses of ZEA ≤ 50 μM did not affect cell viability in LPS-activated human macrophages, indicating that inhibitory effects of ZEA on the production of TNF-α and IL-6 by LPS-activated human macrophages were not caused by dying effects (Figure 6c). For the NLRP3 inflammasome-derived IL-1β secretion, the cells were pretreated with ZEA for 1 h, and then treated with 1 μg/mL LPS for 5 h following ATP (5 mM) or nigericin (10 μΜ) treatments for 30 min. The secretion of IL-1β was detected using ELISA. Our experimental results indicated that ZEA decreased LPS/ATP- and LPS/nigericin-activated IL-1β secretion by human macrophages (Figure 6D,E).

### 2.6. ZEA Reduces the Secretion of Proinflammatory Mediator and Cytokines by LPS-Activated Murine Peritoneal and Bone Marrow-Derived Macrophages (BMDMs)

**Figure 7:**
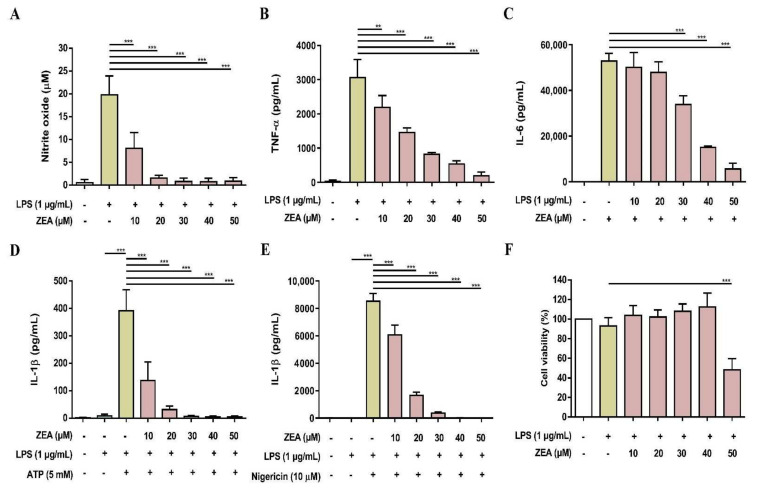
The effect of ZEA on the production of NO and proinflammatory cytokines by LPS-activated murine peritoneal macrophages. The cells were pretreated with ZEA for 1 h, and then treated with 1 μg/mL LPS for 24 h. (**A**) The levels of NO production were analyzed using Griess reaction. Secretions of (**B**) TNF-α and (**C**) IL-6 were detected by ELISA. (**D**,**E**) Murine peritoneal macrophages were pretreated with ZEA for 1 h, and then treated with 1 μg/mL LPS for 5 h following 5 mM ATP or 10 μΜ nigericin treatments for 30 min. The secretion of IL-1β was examined by ELISA. (**F**) Cell viability was detected using MTT assay. Data from three separate experiments are presented as mean ± SD. Statistical significances are presented as ** *p* < 0.01; *** *p* < 0.001.

**Figure 8:**
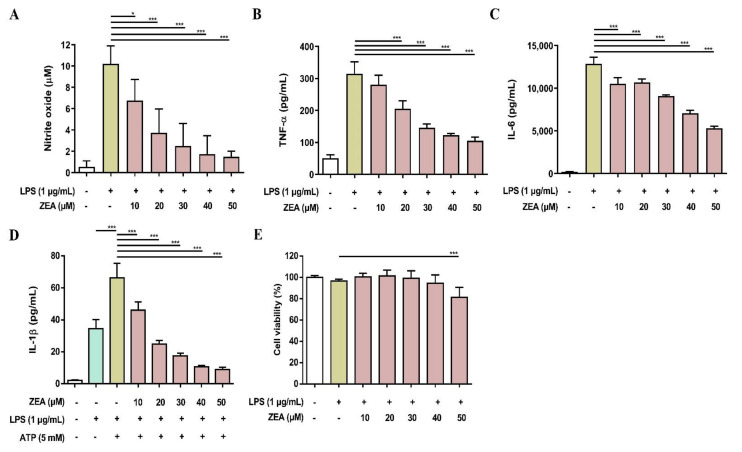
The effect of ZEA on the production of NO and proinflammatory cytokines by LPS-activated BMDMs. The cells were pretreated with ZEA for 1 h, and then treated with 1 μg/mL LPS for 24 h. (**A**) The levels of NO production were analyzed using Griess reaction. Secretions of (**B**) TNF-α and (**C**) IL-6 were detected by ELISA. (**D**) BMDMs were pretreated with ZEA Figure 1. h, and then treated with 1 μg/mL LPS for 5 h following 5 mM ATP for 30 min. The secretion of IL-1β was examined by ELISA. (**E**) Cell viability was detected using MTT assay. Data from three separate experiments are presented as mean ± SD. Statistical significances are presented as * *p* < 0.05; *** *p* < 0.001.

To validate the above cell line results, the inhibitory effects of ZEA on LPS-activated inflammatory responses were further tested using primary cells. Both murine peritoneal macrophages and BMDMs were pretreated with ZEA for 1 h, and then treated with 1 μg/mL LPS for 24 h. The level of NO production was detected using Griess reaction and the secretions of TNF-α and IL-6 were analyzed by ELISA. Cell viability was measured by MTT assay. For the detection of IL-1β secretion, the cells were pretreated with ZEA for 1 h, and then treated with 1 μg/mL LPS for 5 h following ATP (5 mM) or nigericin (10 μM) treatments for 30 min. The levels of IL-1β production were detected using ELISA. As shown in Figure 7, ZEA suppressed LPS-induced NO, TNF-α, IL-6, IL-1β in murine peritoneal macrophages, while the inhibitory doses of ZEA ≤ 40 μM had no cytotoxic effect but 50 μM ZEA revealed cell toxicity. On the other hand, the results also demonstrated that ZEA decreased LPS-induced NO, TNF-α, IL-6, IL-1β in BMDMs. The inhibitory doses of ZEA ≤ 40 μM had no cytotoxic effect but 50 μM ZEA also revealed cell toxicity (Figure 8).

## 3. Discussion

The innate immune system has evolved to protect the host from pathogen infection and macrophages are effector cells of the innate immunity that respond to pathogen infection by initiating phagocytosis and the synthesis and release of pro-inflammatory cytokines [26]. People with a weak immunity have a higher risk of experiencing frequent infections and high mortality rate [27]. Mycotoxins are toxic secondary metabolites produced by fungi and found in many agricultural commodities that have unlike toxic effects according to the toxin and concentration and result in immunostimulatory or immunosuppressive effects [18]. ZEA has been known to have toxic effects on reproduction and fertility [28] and induce an estrogenic activity [4], but the effect of ZEA on immunoregulation has not been well investigated. In the present study, the immunoregulatory effects of ZEA on macrophages under LPS stimulation were examined.

During infection, activated macrophages secrete proinflammatory cytokines (e.g., TNF-α, IL-1β and IL-6) and inflammatory mediators (e.g., NO and PGE_2_) to regulate inflammatory responses against pathogens. A previous study showed that ZEA decreases the iNOS expression and NO production by bovine aortic endothelial cells, resulting in vessel dysfunction [29]. Marin et al. also pointed out that ZEA reveals antagonistic effects on inflammation by decreasing IL-1β and TNF-α expressions in a human hepatocellular carcinoma cell line, HepG2 [30]; additionally, daily intake of ZEA also decreases the serum level of TNF-α in mice [31]. Our experimental results indicated that ZEA attenuates the activities of macrophage upon LPS stimulation, including decreasing NO and PGE_2_ productions and suppressing TNF-α and IL-6 secretions by LPS-activated macrophages. Taken together, these results indicate that ZEA suppresses the LPS-activated immune response in macrophages by decreasing proinflammatory mediator and cytokine productions.

TLR4 stimulation by LPS triggers downstream signaling cascades including MAPKs and NF-κB pathways that are critical in the development of the immune system and regulation of inflammatory and acute immune responses [9]. A previous study indicated that the administration of a ZEA-contaminated diet in weaned pigs for 18 days affects the gene expression of immune regulators, MAPKs, and NF-κB in spleen cells [32]. They found that ZEA increases pro-inflammatory cytokine expression and synthesis, including TNF-α, IL-1β, IL-6 and IL-8, and promotes JNK pathway activation, whereas the activation of *p*-38MAPK and NF-κB is decreased [32]. In addition, Pistol et al. pointed out that ZEA is a potential hepatotoxin, which reduces NF-κB1 and TAK1/p38α MAPK gene expressions and decreases the production of TNF-α, IL-1β, IL-6, IL-8, and IFN-γ in the liver of the experimentally intoxicated piglets [33]. In the present study, the experimental results indicated that ZEA inhibited the phosphorylation of JNK and p38 and attenuated the activation of NF-κB by LPS-activated macrophages, suggesting that ZEA suppresses the LPS-activated immune response in macrophages through attenuating the JNK, p38, and NF-κB signaling pathways.

NLRP3 is a critical intracellular Nod-like receptor that is involved in the recognition of microbial or danger signals and mediates NLRP3 inflammasome assembly, resulting in the maturation and secretion of the pro-inflammatory cytokines, IL-1β and IL-18 [11]. In the present study, the NLRP3 inflammasome activation in macrophages was induced by LPS/ATP and LPS/nigericin, thereby inducing the secretion of IL-1β, enhancing the expression of cleaved caspase-1 and cleaved IL-1β, and increasing the colocalization of ASC and caspase-1, whereas ZEA significantly attenuated the activation of NLRP3 inflammasome by LPS/ATP- and LPS/nigericin-activated macrophages through decreasing the secretion of IL-1β, suppressing the expression of cleaved caspase-1 and cleaved IL-1β, and reducing the colocalization of ASC and caspase-1. These results suggest that ZEA might diminish the activation of NLRP3 inflammasome in macrophages during bacterial infection.

## 4. Conclusions

These experimental results demonstrated that mycotoxin ZEA attenuates innate immune responses by decreasing the production of proinflammatory mediators (NO and PGE_2_) and cytokines (TNF-α, IL-1β and IL-6) by LPS-activated macrophages and inhibiting LPS-activated signaling cascades, including JNK, p38 and NF-κB signaling pathways. Moreover, mycotoxin ZEA also suppresses NLRP3 inflammasome activation by LPS-activated macrophages. Since people with a weak immunity have a higher risk of experiencing frequent infections and severe symptoms, these results suggest that an intake of mycotoxin ZEA-contaminated food might result in decreasing innate immunity, which poses a higher risk of adverse effects during infection.

## 5. Materials and Methods

### 5.1. Animals 

Female C57BL/6 mice (six- to eight-week-old) were obtained from National Lab Animal Center (Taipei, Taiwan) and were kept in pathogen-free facility. All animal handling and experiments were permitted by the Institutional Animal Care and Use Committee at Kaohsiung Medical University (Permit Number: 108101; Period of Protocol: Valid from 1 August 2020 to 31 July 2023). 

### 5.2. Cell Culture 

The murine macrophage cell line (J774A.1), murine fibroblast cell line (L-929), and human THP-1 monocytic cell line were purchased from Bioresource Collection and Research Center (Hsinchu, Taiwan) and cultured in the complete RPMI-1640 medium (Corning, Corning, NY, USA), contained with heat-inactivated fetal bovine serum (10%, Corning), penicillin (100 U/mL, Corning), and streptomycin (100 U/mL, Corning) in a humidified chamber (Binder, Tuttlingen, Germany) at 37 °C. For the differentiation of the THP-1 monocyte into macrophage, the cells were treated with phorbol 12-myristate 13-acetate (PMA, 50 ng/mL) for 24 h at 37 °C in 5% CO_2_.

### 5.3. Peritoneal Macrophags and BMDMs Preparation 

The isolation of thioglycollate-elicited peritoneal macrophages was done following the method of Hung et al. [34] as described previously. Murine bone marrow cells were isolated by the method of Liu et al. [35] as described previously and macrophage differentiation was induced by treating L929 cell-conditioned medium (contained granulocyte-macrophage colony-stimulating factor) for a week following previous studies [36,37]. 

### 5.4. Cell Viability Assay 

The cells (1 × 10^5^) was seeded on a 96-well plate with 200 μL RPMI-1640 medium in each well, and the plate was incubated overnight. Afterwards, cells were pre-treated with 0 ~ 50 µM ZEA (purity ≥ 98%, ChemFaces, Wuhan, Hubei, China) for 1 h following 1 µg/mL LPS treatment (from *E. coli* O111:B4, Sigma Aldrich, St. Louis, MO, USA) for 24 h. Afterwards, the cells were incubated with MTT reagent (5 mg/mL, Sigma Aldrich) for 4 h at 37 °C, followed by 10 min of incubation with stop solution (100 µL isopropanol/0.04 M HCl). The absorbance at 570 nm was detected using a microplate reader (Epoch 2, BioTek Instruments Inc., Winoosky, VT, USA).

### 5.5. NO Production Assay 

The NO assay was followed previously with slight modification [34]. The cells (1 × 10^5^) was seeded on a 96-well plate with 200 μL RPMI-1640 medium overnight. Afterwards, the cells were pre-treated with 0 ~ 50 µM ZEA for 1 h following 1 µg/mL LPS treatment for 24 h. The cell supernatant was harvested and analyzed using Griess reagent (Sigma Aldrich). The absorbance at 540 nm was detected using a microplate reader. The quantity of nitrite was calculated from a sodium nitrite standard curve.

### 5.6. Western Blot Analysis 

J774 A.1 macrophage cells (5 × 10^5^ cells/well) were seeded in 6-well plates overnight. Afterwards, the cells were pre-treated with 0 ~ 50 µM ZEA for 1 h following 1 µg/mL LPS treatment for 2 or 24 h. For the detection of NLRP3 inflammasome-associated protein expression, the cells were pre-treated with different doses (25 and 50 µM) of ZEA for 1 h, and then treated with 1 µg/mL LPS for 5 h following 5 mM ATP treatment for 30 min. After treatments, the cells were washed twice with cold PBS and harvested using RIPA buffer, contained with protease inhibitors (Sigma Aldrich) and phosphatase inhibitors (Fivephoton Biochemicals, San Diego, CA, USA). The protein concentration was quantified by BCA protein assay (Thermo Scientific, Rockford, IL, USA), and equal amounts of proteins were separated by sodium dodecyl sulfate-polyacrylamide gel electrophoresis (SDS-PAGE), and then transferred onto a polyvinylidene fluoride (PVDF) membrane. Afterwards, the PVDF membrane was blocked by 5% non-fat milk/Tris-buffered saline containing 0.05% Tween-20 (TBST), incubated with primary antibodies (Supplemental Table S1), washed by TBST for three times, and then incubated with horseradish-conjugated secondary antibody (Santa Cruz). Then, the PVDF membrane was incubated with ECL chemiluminescence substrate (Thermo Fisher Scientific), and the signals were captured and quantified using a gel imaging system (Bio-Rad Laboratories Inc., Hercules, CA, USA).

### 5.7. qPCR 

The total RNA of J774A.1 cells was extracted using TRIzol reagent and cDNA was generated by SuperScript VILO cDNA synthesis kit (Thermo Fisher Scientific, Rockford, IL, USA). Afterwards, qPCR was performed using SYBR Green PCR Master Mix (Thermo Fisher Scientific) by StepOne Plus Real-Time PCR system (Thermo Fisher Scientific). The primer sequences used in the present study were as follows: *NOS2* forward (F), 5′- GTTCTCAGCCCAACAATACAAGA-3′ and reverse (R), 5′-GTGGACGGGTCGATGTCAC-3′; *COX2* F, 5′-CAAATCCTTGCTGTTCCCACCCAT-3′ and R, 5′-GTGCACTGTGTTTGGAGTGGGTTT-3′; *TNF-α* F, 5′-CAGGCGGTGCCTATGTCTC-3′ and R, 5′-CGATCACCCCGAAGTTCAGTAG-3′; *IL-6* F, 5′-CTGCAAGAGACTTCCATCCAG-3′ and R, 5′-GTGGTATAGACAGGTCTGTTGG-3′; *18S rRNA* F, 5′-CGACGACCCATTCGAACGTCT-3′ and R, 5′-CTCTCCGGAATCGAACCCTGA-3′. Experimental Ct values were normalized to *18S rRNA* and relative mRNA expression calculated versus untreated control sample.

### 5.8. ELISA 

The cells (1 × 10^5^) were seeded on 96-well plates and incubated overnight. Subsequently, the cells were pre-treated with 0 ~ 50 µM of ZEA for 1 h following 1 µg/mL LPS treatment for 24 h. The levels of the TNF-α, IL-6 and PGE_2_ secreted in the cell culture supernatants were analyzed by ELISA kits (Thermo Fisher Scientific) following the manufacturer’s instructions. For the IL-1β secretion, were pretreated with ZEA for 1 h, and then treated with 1 μg/mL LPS for 5 h following 5 mM ATP or 10 μΜ nigericin treatments for 30 min.

### 5.9. NF-κB Promoter Reporter Assay 

J-blue is a J774A.1 subline that stably expresses an NF-kB-inducible SEAP as described previously [34]. The cells were grown in a 96-well plate at a density of 1 × 10^5^ cells/well and incubated overnight. Subsequently, the cells were pre-treated with 0 ~ 50 µM ZEA for 1 h following 1 µg/mL LPS treatment for 24 h. Briefly, Culture supernatant (20 µL) was mixed with QUANTI-blue medium (200 µL, InvivoGen, San Diego, CA, USA), incubated for 45 min at 37 °C, and then the absorbance at 655 nm was detected using a microplate reader.

### 5.10. Immunofluorescence Staining 

J774A.1 cells (1 × 10^5^ cells/well) were seeded on 8-well μ-Slide overnight (ibidi GmbH, Munich, Germany). Afterwards, the cells were pre-treated with various concentrations (25 and 50 µM) of ZEA for 1 h, and then treated with 1 µg/mL LPS for 5 h following 5 mM ATP or 10 μΜ nigericin treatments for 30 min. The cells were washed briefly with PBS and fixed by 4% paraformaldehyde, permeabilized by 0.1% Triton-X 100, incubated with primary antibodies (Supplemental Table S1), washed by PBS, incubated with the secondary antibodies, and then stained nuclei by DAPI (Invitrogen, Carlsbad, CA, USA). The cells were examined using a confocal laser microscope (Leica, Exton, PA, USA) and the images were further analyzed by the Imaris 8 Image Analysis Software (Oxford Instruments, Oxford, UK).

### 5.11. Statistical Analysis

Data from three separate experiments were presented as mean ± SD and the significant differences were evaluated by one-way ANOVA followed by Tukey post-hoc test using GraphPad Prism software version 9 (GraphPad Software, San Diego, CA). Statistical significances are presented as * *p* < 0.05; ** *p* < 0.01; *** *p* < 0.001.

## Data Availability

Data will be provided on request.

## Supplementary Materials

The following are available online at https://www.mdpi.com/article/10.3390/toxins13090593/s1, Table S1: List of primary antibodies used in this study.
