# Supplementary material for: Mycotoxin Zearalenone Attenuates Innate Immune Responses and Suppresses NLRP3 Inflammasome Activation in LPS-Activated Macrophages
Source: Toxins (Basel). 2021 Aug 25;13(9):593. doi: 10.3390/toxins13090593 (PMC8473227; doi:10.3390/toxins13090593)
Supplement: Supplementary file 1 [file toxins-13-00593-s001.zip › toxins-1335866-supplementary.pdf]

# Supplementary Materials: Mycotoxin Zearalenone Attenuates Innate Immune Responses and Suppresses NLRP3 Inflammasome Activation in LPS-Activated Macrophages

**Po-Yen Lee, Ching-Chih Liu, Shu-Chi Wang, Kai-Yin Chen, Tzu-Chieh Lin, Po-Len Liu, Chien-Chih Chiu, I-Chen Chen, Yu-Hung Lai, Wei-Chung Cheng, Wei-Ju Chung, Hsin-Chih Yeh, Chi-Han Huang, Chia-Cheng Su, Shu-Pin Huang and Chia-Yang Li**

**Table S1.** List of primary antibodies used in this study.

| Antibodies | Catalog Number | Company |
|---|---|---|
| Nitric oxide synthase (iNOS) | SC-651 | Santa Cruz Biotechnology (Santa Cruz, CA) |
| Cyclooxygenase 2 (COX-2) | SC-166475 | Santa Cruz Biotechnology |
| Phospho-ERK1/2 | CST#4370 | Cell Signaling (Farmingdale, NY) |
| ERK1/2 | CST#4695 | Cell Signaling |
| Phospho-JNK 1/2 | CST#4668 | Cell Signaling |
| JNK 1/2 | CST#9258 | Cell Signaling |
| Phospho-p38 MAPK | CST#4511 | Cell Signaling |
| p38 MAPK | CST#8690 | Cell Signaling |
| Cleaved caspase-1 | CST#4199 | Cell Signaling |
| Caspase-1 p10 | SC-514 | Santa Cruz Biotechnology |
| Cleaved IL-1β | CST#52718 | Cell Signaling |
| IL-1β | SC-7884 | Santa Cruz Biotechnology |
| ASC | CST#13833 | Cell Signaling |
| NLRP3 | TA336883 | OriGene (Rockville, MD) |
| β-actin | MA5-15739 | Invitrogen (Carlsbad, CA) |
